# The Goldilocks Day for healthy adiposity measures among children and adolescents

**DOI:** 10.3389/fpubh.2023.1158634

**Published:** 2023-09-28

**Authors:** Charlotte Lund Rasmussen, Aleš Gába, Tyman Stanford, Jan Dygrýn, Dorothea Dumuid, David Janda, Karel Hron

**Affiliations:** ^1^Faculty of Physical Culture, Palacký University Olomouc, Olomouc, Czechia; ^2^Department of Public Health and Nursing, Norwegian University of Science and Technology, Trondheim, Norway; ^3^School of Allied Health, Curtin University, Perth, WA, Australia; ^4^Alliance for Research in Exercise, Nutrition, and Activity, Allied Health and Human Performance, University of South Australia, Adelaide, SA, Australia; ^5^Department of Mathematical Analysis and Applications of Mathematics, Palacký University Olomouc, Olomouc, Czechia

**Keywords:** physical activity, sedentary behavior, sleep, time-use, adiposity prevention

## Abstract

**Background:**

The optimal balance of time spent on daily movement behaviors (“The Goldilocks Day”) associated with childhood obesity remains unknown.

**Objective:**

To estimate the optimal durations of sleep, sedentary behavior (SB), light physical activity (LPA), and moderate-to-vigorous physical activity (MPVA) associated with excess adiposity in a paediatric population.

**Methods:**

Accelerometer-measured 24-h movement behaviors were obtained from 659 Czech children and adolescents (8-18-year-olds). Adiposity indicators were body mass index *z*-score, fat mass percentage, fat-free mass index, and visceral adipose tissue. Excess adiposity was defined as exceeding the 85th percentile for an adiposity indicator. Compositional regression analyses were used investigate the associations between movement behaviors and adiposity indicators and estimating “The Goldilocks Day.”

**Results:**

The movement behavior composition was associated with visceral adipose tissue (*F_df1 = 3,df2 = 317_* = 3.672, *p* = 0.013) and fat mass percentage (*F_df1 = 3,df2 = 289_* = 2.733, *p* = 0.044) among children and adolescents. The Goldilocks Day consisted of 8.5 h of sleep, 10.8 h of SB, 3.9 h of LPA, and 0.8 h of MVPA among children and 7.5 h of sleep, 12.4 h of SB, 3.6 h of LPA, and 0.5 h of MVPA among adolescents.

**Conclusion:**

Optimizing the time spent sleeping, and in sedentary and physical activities appears to be important in the prevention of excess adiposity.

## Graphical abstract

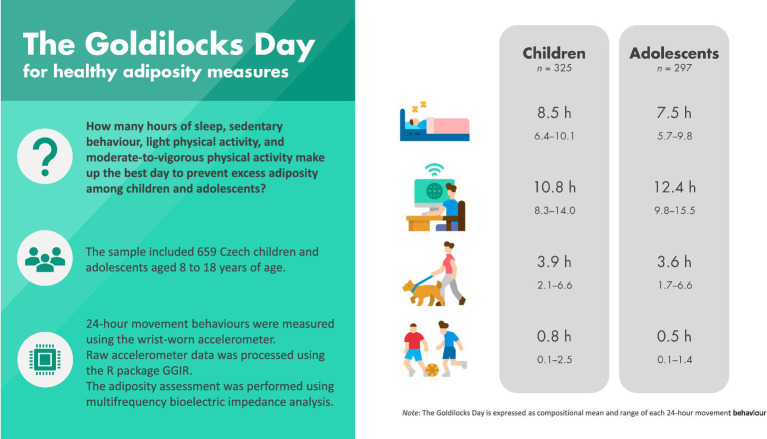

## 1. Introduction

Daily movement behaviors are closely linked to childhood obesity (1). For example, more time spent in moderate-to-vigorous physical activity (MVPA) has consistently been associated with reduced adiposity, whilst unfavorable associations have been reported for excessive sedentary behavior (SB) and short sleep duration (2–5). The World Health Organization (WHO) recommend children and adolescents should perform at least an average of 60 min of MVPA per day and to limit the amount of time spent in SB, particularly the amount of recreational screen time (6). The Canadian Society for Exercise Physiology has taken the guidelines a step further by providing 24-h behavior guidelines recommending children and adolescents to accumulate at least 60 min MVPA, 8–11 h of sleep, and no more than 2 h per day of screen time (7).

Although current guidelines provide recommendations for multiple or all movement behaviors within a day, it is acknowledged that these guidelines are based on studies that do not consider the combined effect of 24-h movement behaviors on health (8, 9). Furthermore, the few studies that do investigate the relationship between 24-h movement behaviors and health rarely describe the optimal durations of each behavior. Instead, they typically investigate how a change in time spent in one behavior at the expense of time spent in remaining behaviors is related to health (8, 10, 11). To the best of our knowledge, only two studies (based on the same sample of Australian school-aged children) have investigated the optimal day, the so-called Goldilocks Day, associated with healthy adiposity among children and adolescents (12, 13). The studies found the optimal day for children’s adiposity consisted of 10.9 h of sleep, 10.8 h of sedentary time, 1.0 h of LPA, and 1.2 h of MVPA (13). Further research on this topic using other datasets is required to strengthen our understanding of optimal the 24-h movement behavior durations for the prevention of childhood obesity.

The aim of this study was to investigate the relationship between 24-h movement behaviors and adiposity indicators in a different population, Czech children and adolescents. Additionally, we aimed to determine the daily durations of sleep, SB, LPA, and MVPA associated with healthy adiposity measures among this population.

## 2. Materials and methods

### 2.1. Participants

Children (8–13 years) and adolescents (14–18 years) were recruited from 11 elementary and secondary schools. Schools with a specific focus on sport and schools for pupils with special educational needs were not included. Children and adolescents, herein collectively referred to as participants, were recruited to participate on a voluntary basis via information flyers that were distributed through the school staff after the school management approved the research. The main inclusion criteria were participant age and good health condition. Participants who reported medical complications that could affect their movement behaviors were excluded from study.

A total of 907 children and adolescents were recruited, of which 248 were excluded either because they withdrew from the study or became ill (*n* = 45), data could not be processed because of technical failures (*n* = 17), provided incomplete sleep logs (*n* = 20), did not meet the accelerometer wear time criteria (*n* = 37), or had incomplete data of the 24-h movement behaviors or adiposity measures (*n* = 129).

### 2.2. Data collection

Data were collected from 2018 to 2019 during regular school weeks. Participants and/or their parents or guardians were asked to fill in questionnaires. Participants and/or their parents or guardians were asked to fill in the questionnaires. Parents or guardians responded to family characteristic questions, while children above 12 years old responded to the remaining items. Parents reported for participants under the age of 12. After 8 days, accelerometers, sleep logs, and questionnaires were collected. The study was approved by the Ethics Committee of the Faculty of Physical Culture, Palacký University Olomouc (reference number: 19/2017).

### 2.3. Measurements of 24-h movement behaviors

Sleep, SB, LPA, and MVPA were measured with wGT3X-BT and GT9X Link ActiGraph accelerometers (ActiGraph, Pensacola, FL, United States) worn by participants on their non-dominant wrist for 24-h over seven consecutive days. These accelerometers are optimal tools for measuring 24-h movement behaviors in children and adolescents due to their high feasibility, reliability, and validity (14, 15). The devices were initialized using the ActiLife software version 6.13.3 (ActiGraph, Pensacola, FL, United States) and sampling interval was set to 100 Hz. Participants were instructed to remove the device only for swimming and bathing.

Categorization of 24-h movement behaviors were determined in accordance with previously published studies (16, 17) using the R package *GGIR* version 1.10–7 (18). Briefly, time spent in SB, LPA, and MVPA were estimated using Hildebrand’s cut points for the Euclidian Norm Minus One metric (19, 20). Sleep duration (i.e., difference between sleep onset and waking up time) was calculated using the heuristic van Hees algorithm guided by the participants’ sleep logs (21). Only data from participants who had worn the accelerometer for at least 16 h/day for at least 4 days (including one weekend day) were included in the analyses (15). Average time spent in sleep, SB, LPA, and MVPA were computed for weekdays and weekend days, and the average daily estimates were calculated as [(average of weekdays × 5) + (average of weekends × 2)]/7.

### 2.4. Adiposity indicators and anthropometric measurements

Body mass index (BMI) was calculated from measured height and weight and expressed as a *z*-score using the WHO reference data to account for sex- and age-related differences (22). Adipose tissue was measured by multifrequency bioelectric impedance analysis (InBody 720; Biospace Co., Ltd., Seoul, Korea) using manufacturer’s recommended equations to estimate the percentage of fat mass (FM%) and visceral adipose tissue (VAT). Fat mass index (FMI) was calculated by dividing the amount of fat mass (kg) by height squared.

Body height and weight were measured using standardized procedures with participants wearing light clothing while barefoot. Body height was measured before adiposity assessment using the research grade anthropometer P-375 (Trystom, Olomouc, Czech Republic) with a precision of 0.1 cm. Body weight was measured by the body composition analyser with an accuracy of 0.1 kg. The participants were given instructions to abstain from eating for a minimum of 4 h and to ensure that they were properly hydrated for at least 24 h prior to the examination. Measurements were carried out in the morning during school hours.

The adiposity assessment using the InBody 720 device is considered to be sufficiently precise as it was validated against dual-energy X-ray absorptiometry in the target population (23).

### 2.5. Covariates

We considered sex, maternal BMI, maternal education, and unhealthy snacking as potential confounders based on theoretical assumptions and previous literature (24–26). Maternal BMI was calculated from self-reported body height and weight. Maternal education was based on self-reported highest educational level and was dichotomized before analysis (0 = lower than university level, 1 = university level). Information on unhealthy snacking was obtained by the question, “About how many times a week do you usually eat or drink (1) sweets (candy or chocolate), (2) coke or other soft drinks that contain sugar, and (3) crisps, chips, salt sticks, etc.?” with the following response categories: “never,” “less than once a week,” “once a week,” “2–4 times a week,” “5–6 times a week,” “once a day (everyday),” or “more than once every day.” Unhealthy snacking was classified as consuming at least one of the unhealthy snacks at least once a day.

### 2.6. Statistical analysis

Standard descriptive statistics were used to present the characteristics of the study population in terms of means and standard deviations or as frequencies and percentages. For each participant, 24-h time-use was partitioned into time spent in sleep, SB, LPA, and MVPA (i.e., a four-part movement behavior composition). There were no zeros in any of the measured behavior variables. Compositional means were used to describe the 24-h time-use composition, calculated as the geometric means for each compositional part, adjusted to sum to 24 h.

Each participants’ time-use composition was expressed as a set of three isometric log-ratio (*ilr*) coordinates (27). Next, the relationship between movement behavior composition and adiposity indicators were investigated using compositional linear regression models, that is, by regressing the adiposity indicators (dependent variable) on the *ilrs* (independent variables). Models were stratified by age (i.e., separate models for children and adolescents) and included adjustment for sex, maternal BMI, maternal education, and unhealthy snacking. A total of 37 participants had missing information on either maternal education or maternal BMI. We assumed these missing values to be missing completely at random (MCAR), and thus performed a complete case analysis by excluding those with missing values (28). We used the Type III analysis of variance *F*-test to assess if 24-h time-use composition was associated with each of the adiposity measures (i.e., significance of set of *ilr*s after adjustment for covariates) (29). If a statistically significant relationship between the 24-h time-use composition and an adiposity indicator was detected (*p* < 0.05), optimal time-use compositions were estimated following the approach described in (12).

The multiple linear regression models were used to predict adiposity indicators for every 24-h movement behavior composition within the empirical data to avoid extrapolation out of the range of sampled data. Adiposity outcomes associated with these 24-h time-use compositions, as estimated by the compositional regression models, were plotted in 3D quaternary plots. The “optimal” 24-h time-use composition (i.e., Goldilocks Day) was defined as the compositional mean of all compositions that were associated with healthy adiposity status. Based on a review of previous literature (30) and the distribution in the predicted adiposity indicators, values below the 85th percentile of specific adiposity indicator were considered as healthy adiposity measures. Thus, the Goldilocks Day represented the amount of time spent in each behavior that was associated with healthy adiposity measures.

Analyses were performed in R version 4.1 (31), using the *compositions* (32) and *car* (29) packages.

### 2.7. Sensitivity analysis

We investigated the potential confounding effect of wear time by including the log of total wear time (as measured by the accelerometer) in the adjusted models. Differences between age-groups and between participants with and with-out missing values (Supplementary Table S1) were investigated by calculating means and standard deviations or frequencies and percentages. Group differences in characteristics were tested using *t*-test, Chi-squared or Kruskal-Wallis statistics, whereas differences in compositional means were tested using MANOVA.

### 2.8. Availability of data and materials

The dataset analysed during the current study is available in the Figshare repository, https://doi.org/10.6084/m9.figshare.20553108.v1.

## 3. Results

The overall and age-stratified characteristics of the study participants are shown in Table 1. In the total sample, mean age was 13.9 ± 2.8 years; mean BMI *z*-score was 0.22 ± 1.07; almost half of the sample were boys (42.5%); more than half consumed unhealthy snacks more than once/day (66.2%); and the 24-h compositional mean consisted of 8.1 h sleeping time, 11.3 h of SB, 3.8 h of LPA time and 0.8 h of MVPA time. Differences between age groups were observed for FMI, VAT, unhealthy snacking, and all components of 24-h time-use composition (*p* < 0.001 for all). There were no differences in descriptive characteristics between participants with and without missing data (Supplementary Table S1).

**Table 1:** Characteristics of the study participants stratified by age-groups.

	Overall (*N* = 659)	Children (*n* = 343)	Adolescents (*n* = 316)	Value of *p*^d^
	Mean (SD) or count (%)	Mean (SD) or count (%)	Mean (SD) or count (%)	
Age (years)	13.9 (2.8)	11.7 (1.6)	16.3 (1.3)	<0.001
BMI *z*-score	0.22 (1.07)	0.25 (1.14)	0.20 (0.99)	0.557
Fat mass (%)	20.1 (8.7)	19.5 (8.3)	20.8 (9.1)	0.094
Fat mass index (kg/m^2^)	4.3 (2.5)	3.8 (2.2)	4.7 (2.7)	<0.001
Visceral adipose tissue (cm^2^)	48.8 (31.2)	43.1 (28.1)	55.0 (33.2)	<0.001
24-h movement behaviors composition (hours/day)^a^				<0.001
Sleep	8.1	8.6	7.5	
Sedentary behavior	11.3	10.4	12.2	
Light PA	3.8	4.0	3.6	
Moderate-to-vigorous PA	0.8	1.0	0.7	
24-h movement behaviors composition hours/day (SD)^b^				
Sleep	8.1 (0.9)	8.7 (0.6)	7.7 (0.7)	<0.001
Sedentary behavior	11.4 (1.6)	10.6 (1.4)	12.4 (1.3)	<0.001
Light PA	3.9 (0.8)	4.0 (0.7)	3.7 (0.8)	<0.001
Moderate-to-vigorous PA	0.8 (0.4)	1.0 (0.4)	0.9 (0.3)	<0.001
Wear time (hours/day)	24.3 (0.6)	24.3 (0.6)	24.4 (0.7)	<0.001
Sex				
Boys^c^	280 (42.5%)	147 (42.9%)	133 (42.1%)	0.904
Maternal BMI (kg/m^2^)	24.4 (4.1)	24.5 (4.1)	24.3 (4.1)	0.339
Missing^c^	33 (5.0%)	17 (5.0%)	16 (5.1%)	
Maternal education^c^				0.589
Lower than university	379 (57.5%)	193 (56.3%)	186 (58.9%)	
University	264 (40.1%)	141 (41.1%)	123 (38.9%)	
Missing	16 (2.4%)	9 (2.6%)	7 (2.2%)	
Unhealthy snacking^c^				<0.001
Low frequency	223 (33.8%)	143 (41.7%)	80 (25.3%)	
High frequency	436 (66.2%)	200 (58.3%)	236 (74.7%)	

BMI, body mass index. PA, physical activity. SD, standard deviation. ^a^Compositional mean. ^b^Aritmetric means. ^c^The number and proportion of participants within each corresponding category. Unhealthyh scnaking defined as consuming at least one of the unhealthy snacks at least once a day. ^d^Differences between children and adolescents were tested using the *t*-test for independent samples, Chi-squared test, Kruskal-Wallis test, or multivariate analysis of variance based on the type and normality of variable.

### 3.1. Relationship between 24-h movement behavior composition and adiposity indicators

Associations between the overall 24-h time-use composition and adiposity indicators are displayed in Table 2. Among children, VAT was associated with the 24-h composition (*F_3,317_* = 3.672, *p* = 0.013). Fat mass percentage was related to the 24-h composition among adolescents (*F_3,289_* = 2.733, *p* = 0.044). Including wear time in the adjusted models did not alter the results (not shown).

**Table 2:** Associations between overall 24-h time-use composition and adiposity measures, stratified by age-group.

	Children (*n* = 325)		Adolescents (*n* = 297)	
	*F* (*df1, df2*)	Value of *p*	*F* (*df1, df2*)	Value of *p*
BMI *z*-score	0.568 (3, 317)	0.637	0.352 (3, 289)	0.788
Fat mass percentage	0.990 (3, 317)	0.398	2.733 (3, 289)	0.044
Fat mass index	1.745 (3, 317)	0.158	1.838 (3, 289)	0.140
Visceral adipose tissue	3.672 (3, 317)	0.013	3.176 (3, 289)	0.091

*F* (df1, df2), F-statistic on degrees of freedom (numerator) and degrees of freedom (denominator), respectively, for the Type III set of 24-h time-use composition ilrs, adjusted for sex, frequency of unhealthy snack, maternal body mass index, and maternal education. Model results are based on complete cases.

### 3.2. Goldilocks day for adiposity indicators

**Figure 1:**
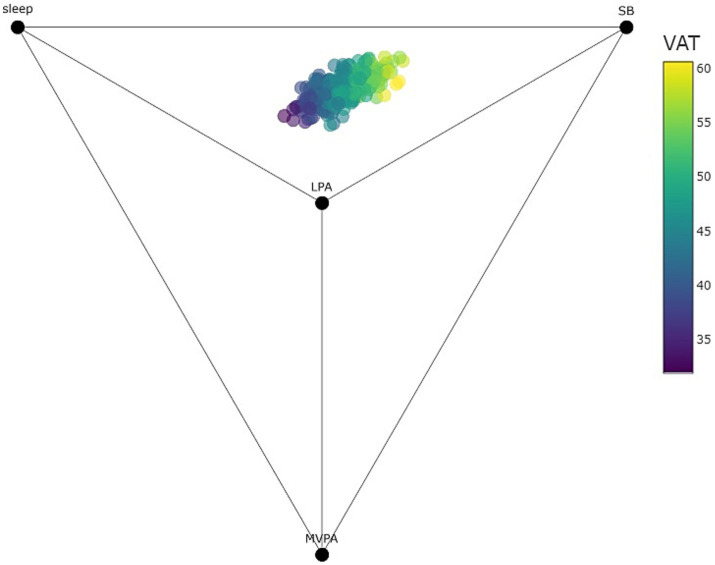
Quaternary diagram (tetrahedron) showing all observed compositions and predicted visceral adiposity tissue (VAT) among children as points within the tetrahedron (points colored by predicted VAT). Note that all 24-h time-use compositions are contained within the tetrahedron. Time-use compositional variables can be observed as the proportional distance of points between their respective labeled vertex and opposing face (for example, the points in the sample are only a fraction of the distance towards the MVPA vertex showing the relatively small amount of time spent in MVPA).

**Figure 2:**
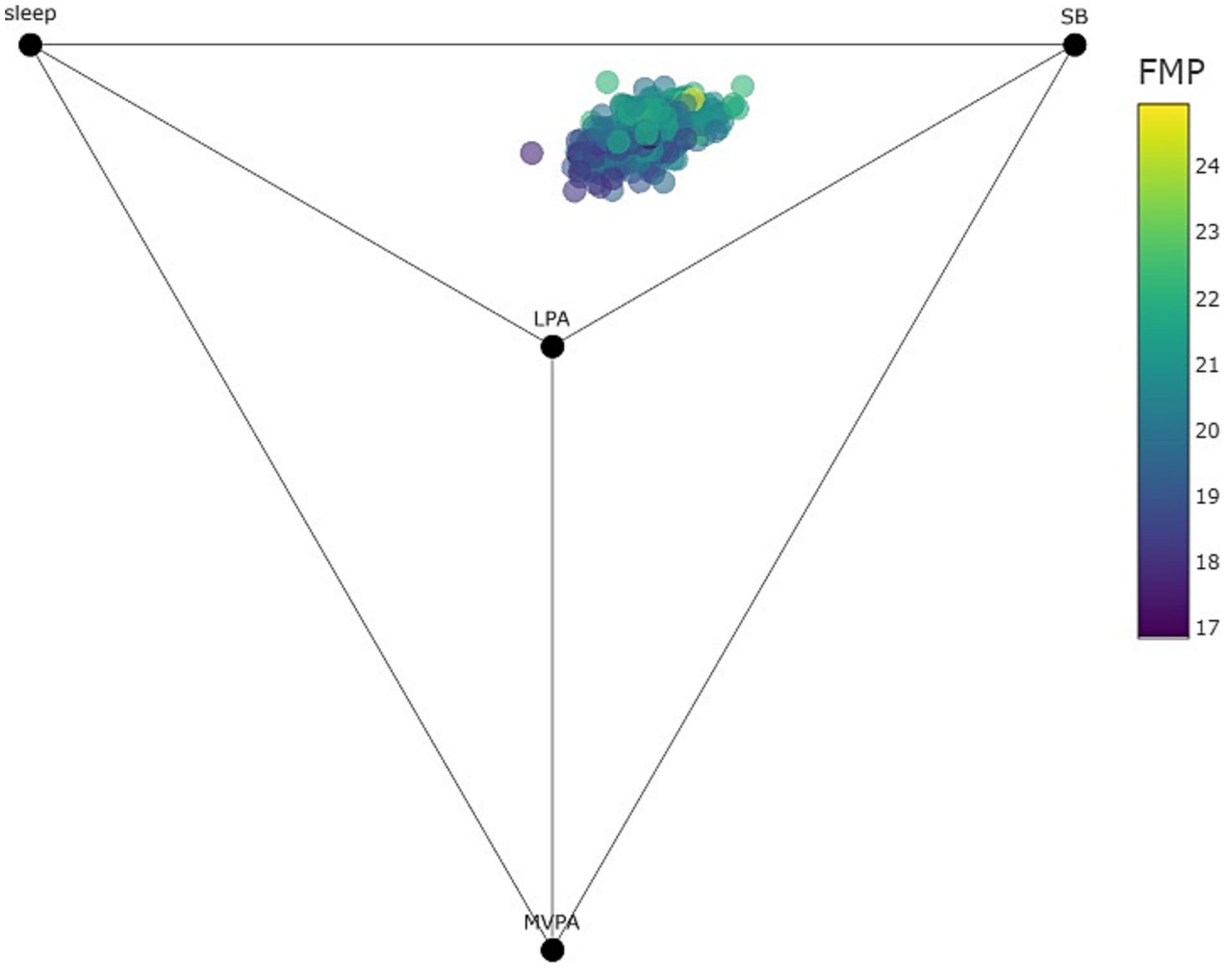
Quaternary diagram (tetrahedron) showing all observed compositions and predicted FM% among adolescents as points within the tetrahedron (points colored by predicted FM%). Note that all 24-h time-use compositions are contained within the tetrahedron. Time-use compositional variables can be observed as the proportional distance of points between their respective labeled vertex and opposing face (for example, the points in the sample are only a fraction of the distance towards the MVPA vertex showing the relatively small amount of time spent in MVPA).

Figures 1 2 are quaternary tetrahedrons showing all compositions observed in the sample of children and adolescents, respectively. Behaviors (i.e., sleep, SB, LPA, and MVPA) are 100% (24 h) at the corresponding apices of the tetrahedron and 0% at the opposite base. Each dot represents a predicted value of the outcome (i.e., VAT in Figure 1 and FM% in Figure 2), with yellow representing high values and blue representing lower values. Thus, both Figures 1 2 shows that 24-h time-use compositions consisting of more sleep, LPA and MVPA and less SB were associated with lower VAT values among children and lower FM% values among adolescents. Interactive versions of Figures 1 2 with hover text and that can be rotated and zoomed in on can be found here: https://tystan.shinyapps.io/czech_goldilocks/.

For children, the centre of the 24-h movement compositions associated with healthy adiposity expressed by VAT (i.e., the Goldilocks Day) consisted of 8.5 h of sleep, 10.8 h of sedentary time, 3.9 h of LPA and 0.8 h of MVPA (Table 3). For adolescents, the centre of the 24-h compositions associated with healthy adiposity expressed by FM% consisted of 7.5 h of sleep, 12.4 h of sedentary time, 3.6 h of LPA ad 0.5 h of MVPA.

**Table 3:** Goldilocks day: optimal 24-h movement behavior composition for adiposity measures, stratified on age-group.

	Children		Adolescents	
	Compositional mean	Range*^b^*	Compositional mean	Range*^b^*
Optimal time use (h/day)				
Sleep	8.5	6.4–10.1	7.5	5.7–9.8
Sedentary behaviour	10.8	8.3–14.0	12.4	9.5–15.5
Light PA	3.9	2.1–6.6	3.6	1.7–6.6
Moderate-to-vigorous PA	0.8	0.1–2.5	0.5	0.1–1.4
Outcome variable^a^	48.1	41.2–60.7	20.5	19.0–25.0

Optimal 24-h movement behaviour composition estimated as the compositional centre of predictive composition associated below the 85th percentile of the outcome measure. PA, physical activity. ^a^Visceral adipose tissue was used for children and fat mass percentage for adolescents. These data are presented by arithmetic mean. ^b^Please note that the ranges of each 24-h movement behavior are strongly dependent and computed here for illustrative purposes.

## 4. Discussion

### 4.1. Main findings

In this study we aimed to investigate the relationship between 24-h movement behaviors and adiposity indicators among children and adolescents. Furthermore, we estimated the Goldilocks Day of time spent in sleep, SB, LPA, and MVPA associated with healthy adiposity measures. Among children, we observed that the 24-h movement behavior composition was associated with VAT. The Goldilocks Day associated with healthy adiposity expressed by VAT consisted of 8.5 h of sleep, 10.8 h of SB, 3.9 h of LPA, and 0.8 h of MVPA. Among adolescents, the 24-h movement behavior composition was related to FM%, where the Goldilocks Day consisted of 7.5 h of sleep, 12.4 h of sedentary time, 3.6 h of LPA ad 0.5 h of MVPA.

### 4.2. Comparison with previous findings

To date, only two studies have investigated optimal 24-h time-use associated with adiposity in the paediatric population (12, 13) both of which used the same study population of 1,182 Australian school-aged children. The authors found that children spending 10.9 h sleeping, 10.8 h sedentary, 1 h on LPA and 1.2 h on MVPA had the best adiposity indicators. These results differ notably from those of the current study, particularly in estimated time that children and adolescents should spent on sleep, LPA and MVPA. Moreover, we did not find a significant relationship between the 24-h movement behavior composition and indicators of total adiposity (i.e., BMI *z*-score and FMI) among children or adolescents. However, comparability between studies is limited primarily due to differences in adiposity indicators, the definition of a “healthy” adiposity status, and study samples. While we used BMI *z*-scores, FM%, FMI, and VAT, Dumuid et al. (13) computed a composite adiposity score consisting of body composition, BMI *z*-score and waist-hip-ratio. Additionally, the difference in age of study samples should be acknowledged, as adiposity and behavior patterns significantly change during the transition from childhood to adolescence (33).

An unexpected finding from our study is that the predicted hours of sleep and MVPA are less than what is recommended by global health authorities, i.e., 9–11 h of sleep for children, 8–10 h of sleep for adolescents and 1 h of MVPA for both (6, 34). A possible explanation for this finding may be the fact that childhood obesity is linked to a wealth of factors (e.g., diet, genetics, and environmental exposures) and thus, not solely 24-h movement behaviors. Accordingly, we found that the 24-h movement behaviors composition only explained ~5% of the variability in adiposity indicators (results not shown). We encourage future studies to investigate the complex interplay between 24-h movement behaviors as well as other modifiable and non-modifiable risk factors for childhood obesity.

Another under-investigated area is the role of LPA in the prevention of childhood obesity. We found that children and adolescents engaging in a little less than 4 h of LPA daily had the best VAT and FM% measures, respectively. Although our results suggest that LPA is an important component of the 24-h movement behavior composition, the overall evidence on the health impact of LPA among children and adolescents is inconclusive. Our results concur with some studies finding LPA to be beneficially associated with adiposity and cardiovascular risk markers in children and adolescents (35–38). However, other studies found no relationship between LPA and adiposity measures among this population group (39, 40). Thus, more research examining the potential of LPA (relative to remaining 24-h movement behaviors) for the prevention of obesity in the paediatric population is warranted.

The wide range of predicted values for each component of the Goldilocks Day indicates that the optimal day might differ between individuals. For example, only 5.7 h of sleeping was associated with healthy adiposity among some adolescents in our sample. At the individual level, it seems that insufficient sleep may be compensated for by other movement behaviors (i.e., reduced SB, increased levels of LPA and MVPA) to achieve a healthy adiposity profile. This variability should be considered for next generation of 24-h movement behavior guidelines. Furthermore, it can be assumed that other lifestyle behaviors (e.g., unhealthy eating habits) contribute to the imbalance in the 24-h movement behavior composition. For example, if a child has a very high energy intake, he/she must increase energy expenditure (e.g., increase PA at the expense of sleep) to achieve a healthy adiposity state. Accordingly, it seems prudent to consider various lifestyle factor when investigating the relationship between 24-h movement behaviors and adiposity indicators.

### 4.3. Practical implications

Today, global health organizations are acknowledging that all behaviors throughout the day have implications for health (41, 42). This was partly supporting by our findings, which showed that “the whole day matters” for the prevention of childhood obesity. Accordingly, it would seem prudent to broaden the focus beyond increasing MVPA when planning strategies and interventions for the prevention of childhood obesity. Instead, we suggest shifting towards a more holistic approach to the assessment of 24-h movement behaviors and public health messaging about their benefits. Nevertheless, it should be acknowledged that more research is required to understand what an optimal 24-h day for health looks like for children and adolescents as this is, to the best of our knowledge, only the third study investing this. Moreover, we only investigated the optimal 24-h day for adiposity indicators, but this might differ depending on which health outcome is considered. Thus, we encourage future work to investigate optimal 24-h behavioral time-use for a range of health outcomes.

### 4.4. Strengths and limitations

The current study has several strengths. Firstly, this is the first study using the Goldilocks approach for estimating the “optimal” 24-h time-use composition associated with adiposity among European children and adolescents. Secondly, the multi-day raw accelerometer data were analysed using open-source R-package, which contributes to the transparency and subsequent reproducibility. Third, the use of compositional data analysis was another strength of this study, enabling the methodologically appropriate investigation of the relationship between all 24-h movement behaviors and adiposity indicators. Lastly, since the previous studies (13, 43) using a similar methodology were conducted on a sample with a narrow age range, the current study is the first attempt to estimate the Goldilocks Day in both children and adolescents.

There were several limitations that should be noted. Given the cross-sectional design, there is a risk of reverse causality between 24-h time-use and adiposity. The accelerometers were worn on the wrist, which may have resulted in an overestimation of the time spent sedentary (44). Another limitation might be that, despite the 24-h movement guidelines encourage avoiding excessive recreational screen time and prolonged sitting (42), the current study analysed total sedentary time without considering the context and patterns of SB. Consequently, direct comparison between our findings and other studies may not be possible. The self-reported information on the potential confounders was a limitation by increasing the risk of residual confounding. Finally, we did not have information on puberty status, which may have confounded the estimated relationship between the 24-h time-use composition and adiposity indicators.

## 5. Conclusion

Balancing time spent on movement behaviors within a day appears to be an appropriate strategy against excess adiposity in the paediatric population. The results of the current study suggests that the whole day matters. If confirmed in future studies, this would indicate that a shift towards a more holistic approach when investigating the role of 24-h movement behaviors in preventing childhood obesity is required. Additionally, we found age difference in what could be considered the Goldilocks Day and a wide range of optimal durations for all components of 24-h time-use which indicate that “one size does not fit all.” Future efforts in developing public health guidelines and prevention strategies should consider an integrated and more personalized approach to improve sleep, SB, and PA of different intensities in children and adolescents.

## Data availability statement

The datasets presented in this study can be found in online repositories. The names of the repository/repositories and accession number(s) can be found at: https://doi.org/10.6084/m9.figshare.20553108.v1.

## Ethics statement

The studies involving humans were approved by the Ethics Committee of the Faculty of Physical Culture, Palacký University Olomouc (reference number: 19/2017). The studies were conducted in accordance with the local legislation and institutional requirements. Written informed consent for participation in this study was provided by the participants’ legal guardians/next of kin.

## Author contributions

CLR, AG, JD, and DJ contributed to the conception and design of the study. CLR and AG wrote the first draft of the manuscript. CLR performed the statistical analysis with guidance from AG, TS, DD, and KH. TS was responsible for generating the virtual figures. All authors contributed to manuscript revision, proof reading and approval of the submitted version.
