# Supplementary material for: The Goldilocks Day for healthy adiposity measures among children and adolescents
Source: Front Public Health. 2023 Sep 28;11:1158634. doi: 10.3389/fpubh.2023.1158634 (PMC10569221; doi:10.3389/fpubh.2023.1158634)
Supplement: Supplementary file 1 [file Table_1.DOCX]

**Supplementary File 1.**

**Table S1.** Baseline characteristics of the participants with and without missing values in maternal BMI and/or maternal education.

|  | Non-missing (*n* = 622) |  | With missing (*n* = 37) |  | *p-value* |
| --- | --- | --- | --- | --- | --- |
|  | Mean (SD) or count (%) |  | Mean (SD) or count (%) |  |  |
| Age (years) | 13.9 (2.8) |  | 14.4 (2.4) |  | 0.216 |
| BMI *z*-score | 0.22 (1.07) |  | 0.48 (1.1) |  | 0.136 |
| Fat mass (%) | 20.1 (8.7) |  | 21.1 (10.0) |  | 0.701 |
| Fat mass index (kg/m^2^) | 4.3 (2.5) |  | 4.8 (3.0) |  | 0.422 |
| Visceral adipose tissue (cm^2^) | 48.8 (31.2) |  | 56.2 (34.6) |  | 0.178 |
|  |  |  |  |  |  |
| 24-hour movement behaviours composition (hours/day)^a^ | | | | | 0.978 |
| Sleep | 8.1 |  | 8.0 |  |  |
| Sedentary behavior | 11.3 |  | 11.4 |  |  |
| Light PA | 3.8 |  | 3.8 |  |  |
| Moderate-to-vigorous PA | 0.8 |  | 0.8 |  |  |
| 24-hour movement behaviours composition (hours/day (SD))^b^ | | | | | |
| Sleep | 8.1 (0.9) |  | 8.1 (0.9) |  | 0.647 |
| Sedentary behavior | 11.4 (1.6) |  | 11.5 (1.7) |  | 0.842 |
| Light PA | 3.9 (0.8) |  | 3.8 (1.0) |  | 0.894 |
| Moderate-to-vigorous PA | 0.8 (0.4) |  | 0.8 (0.4) |  | 0.832 |
| Wear time (hours/day), mean (SD) | 24.3 (0.6) |  | 24.3 (0.7) |  | 0.905 |
|  |  |  |  |  |  |
| Boys^c^ | 280 (42.5%) |  | 17 (45.9%) |  | 0.790 |
| Maternal BMI (kg/m^2^) | 24.4 (4.1) |  | 23.2 (1.7) |  | 0.679 |
| Missing^c^ | 33 (5.0%) |  | 33 (89.2%) |  |  |
| Maternal education^c^ |  |  |  |  |  |
| Lower than university | 379 (57.5%) |  | 11 (29.7%) |  | 0.692 |
| University | 264 (40.1%) |  | 10 (27.0 %) |  |  |
| Missing | 16 (2.4%) |  | 21 (43.2%) |  |  |
| Unhealthy snacking^c^ |  |  |  |  | 0.715 |
| Low frequency | 223 (33.8%) |  | 11 (29.7%) |  |  |
| High frequency | 436 (66.2%) |  | 26 (70.3 %) |  |  |

*BMI = Body mass index. PA = Physical activity. SD = Standard deviation. ^a^Compositional mean. ^b^Aritmetric means.^c^The number and proportion of participants within each corresponding category.P=p-value based on two-sample t-test, Chi-squared test, Kruskall-Wallis test or MANOVA.*
